# Relationship between bronchopulmonary dysplasia phenotypes with high-resolution computed tomography score in early preterm infants

**DOI:** 10.3389/fped.2022.935733

**Published:** 2022-09-20

**Authors:** Qiong Yao, Quan-li Shen, Guo-ying Huang, Xi-hong Hu

**Affiliations:** ^1^Department of Radiology, Children’s Hospital of Fudan University, Shanghai, China; ^2^Cardiac Center, Children’s Hospital of Fudan University, Shanghai, China

**Keywords:** bronchopulmonary dysplasia, preterm infants, pulmonary hypertension, computed tomography, tracheobronchomalacia

## Abstract

**Objective:**

To assess the relationship between high-resolution computed tomography (HRCT) abnormalities and clinical phenotypes of bronchopulmonary dysplasia (BPD).

**Methods:**

A retrospective, single-center study was carried out at the Children’s Hospital of Fudan University between 2013 and 2020. Preterm infants born at ≤ 32 weeks’ gestation who were diagnosed with BPD and had HRCT between 40 and 50 weeks postmenstrual age (PMA)were included in the study. HRCT images from six pulmonary lobes were scored based on seven types of pulmonary lesions from two categories: hyperaeration lesions and parenchymal lesions. The hyperaeration score (HS) included scores of decreased attenuation, mosaic attenuation, and bulla/bleb, while the parenchymal score (PS) included those of linear lesion, consolidation, bronchial wall thickening, and bronchiectasis. All seven scores were summed up to create the total score (TS). One-way ANOVA testing or Kruskal-Wallis testing was adopted for the comparison of HRCT scores with BPD severity and clinical phenotypes. The correlation between HRCT scores and clinical phenotypes was evaluated by Spearman’s correlation analysis.

**Results:**

A total of 81 cases were included in the study. Cases with more severe BPD had a higher TS (*p* = 0.01), HS (*p* = 0.02), PS (*p* = 0.02), mosaic attenuation score (*p* = 0.03), bulla/Bleb score (*p* = 0.03), and linear density score (*p* = 0.01). TS (*r* = 0.28), PS (*r* = 0.35), linear density (*r* = 0.34), and consolidation (*r* = 0.24) were correlated with pulmonary hypertension (PH). However, no HRCT score was significantly different between the patients with or without tracheobronchomalacia (TBM). BPD patients with a combination of lung parenchymal disease, PH, and TBM had the highest TS and HS.

**Conclusion:**

HRCT scores correlated with BPD severity and PH in our study. HS might be a useful tool in the assessment of BPD severity while linear densities and consolidation might be helpful in predicting PH.

## Introduction

Bronchopulmonary dysplasia (BPD) is a respiratory complication frequently present in preterm infants (1). In China, the incidence of BPD in preterm infants with gestational age (GA) ≤ 28 weeks reached up to 47.8% in 2019 (2). With the application of improved respiratory support strategies, antenatal steroids, surfactant therapy, and other advanced clinical technologies, an increasing number of preterm infants have survived, and the pathological process of BPD, therefore, has changed. There is growing interest in the underlying cardiopulmonary disorders and large airway complications (3–5). BPD patients are prone to pulmonary hypertension (PH) due to impaired angiogenesis and alveolarization. PH has a major impact on the prognosis and survival rate of BPD patients (6). Positive pressure respiratory support and endotracheal intubation may deform the immature airway, and thereby bring about large airway lesions, such as tracheobronchomalacia (TBM) (7). Although the current BPD definitions still define BPD and its severity based on the level of respiratory support at 36 weeks’ postmenstrual age (PMA) (8, 9), classification of BPD infants into clinical phenotypes according to their predominant clinical features may facilitate better risk stratification (3).

Chest radiographs, high-resolution computed tomography (HRCT), and magnetic resonance imaging were used to assess the BPD-associated lung abnormalities in previous studies (10–12). Of these methods, HRCT has high accuracy and sensitivity. Numerous researchers have used HRCT to semi-quantify pulmonary structural abnormalities and to predict long-term outcomes of BPD patients (12). However, the relations between HRCT and clinical phenotypes of BPD patients are still unclarified. Existing data suggest that HRCT may be useful in the phenotypic classification of BPD patients and therefore may contribute to clinical decision-making and more accurate follow-ups of these patients. The purpose of this study is to explore the associations between HRCT abnormalities and clinical phenotypes in preterm infants with BPD.

## Materials and methods

### Subjects’ clinical data

A retrospective, single-center study was carried out at the Children’s Hospital of Fudan University between 2013 and 2020. Preterm infants born at ≤ 32 weeks’ gestation who were diagnosed with BPD and had HRCT between 40 and 50 weeks PMA were included in the study. Those with poor HRCT images, congenital pulmonary deformities, serious pulmonary infections, and severe congenital cardiac disease were excluded from the study. Clinical information of the included patients was reviewed *via* the medical records system, including GA, body weight (BW), the duration of respiratory support in the hospital, and the existence of PH and large airway lesions. Other parameters that could affect the development of BPD, such as 1-min and 5-min Apgar scores, the use of surfactant therapy and antenatal steroids, presence of histological chorioamnionitis, and patent ductus arteriosus, were also examined. BPD was defined and its severity was graded by the NHLBI 2018 revision (8).

In this study, HRCT scores, as a representation of the severity of lung parenchymal disease, were assessed in infants with or without clinical phenotypes of PH and large airway disease of TBM. Specifically, the patient is diagnosed with PH if the pulmonary arterial pressure is estimated to be > 35 mmHg by the tricuspid valve jet velocity on echocardiogram (13). TBM is defined as more than 50% of the airway lumen narrowing during expiration under bronchoscopy (14). If HRCT suggested tracheobronchial stenosis, bronchoscopy would be performed to confirm the disease. This study was approved by the Institutional Review Board and consent was obtained from the parents of each patient.

### High-resolution computed tomography protocol

High-resolution computed tomography was conducted using a 64-detector scanner (GE Healthcare, Princeton, NJ, United States) with a tube voltage of 80 kV, a tube current of 60 mAs, and a matrix of 512 × 512. The images were acquired at end-inspiration from the apex of the chest to the diaphragm. All HRCT scans had a reconstruction slice thickness of 0.625 mm. Infants were either sedated with oral chloral hydrate (25 mg/kg) or asleep after feeding. The scanning time of HRCT was 3.5∼5 s.

### High-resolution computed tomography scoring protocol

For HRCT scoring, we adopted an HRCT scoring system for BPD by Sung et al., which was modified on the basis of the HRCT scoring systems used in the recent 10 years (10, 15–17). We evaluated six pulmonary lobes (left upper lobe, left lingual segment, left lower lobe, right upper lobe, right middle lobe, and right lower lobe) and examined seven types of lesions in each lobe to identify the presence of pulmonary abnormalities. Seven types of pulmonary lesions were categorized into two types: hyperaeration and parenchymal lesions. Hyperaeration lesions included decreased attenuation, mosaic attenuation, and bulla/bleb, while parenchymal lesions included linear lesion, consolidation, bronchial wall thickening, and bronchiectasis. The radiographic definitions were defined by the Fleischner Society nomenclature (18). Decreased attenuation was defined as an area of reduced lung attenuation and mosaic attenuation was a non-homogeneous lesion that exhibited various attenuations. Bulla (≥ 1 cm) or bleb (≤ 1 cm) are referred to as round local lesions with reduced attenuation. Consolidation represented a homogeneous increase in parenchymal attenuation with blurred blood vessel and bronchial wall boundaries, linear lesion marked a thin and extended lesion along with soft tissue attenuation, and bronchiectasis stood for a widened airway compared to the accompanying pulmonary blood vessels.

For each lobe, one point was given for the presence of an abnormal lesion of the seven parameters, and 0 points were given if the lesions were not present. The maximum score for each lobe was seven points. The total HRCT score of six lobes was summed, including the hyperaeration score (HS), the parenchymal score (PS), and the total score (TS). Hence, a higher score reflected more severe pulmonary disease.

High-resolution computed tomography images were analyzed independently by two radiologists with more than 5 years of experience in pediatric pulmonary imaging and the scores were averaged for analysis. TS, HS, and PS were compared for inter-observer agreement evaluation. The scans were reviewed by the same observer (Dr. Yao) 1 month later to measure intra-observer agreement.

### Data analysis

Statistical analysis was carried out with SPSS (version 26.0, IBM, Armonk, NY, United States). Continuous parameters were expressed as the mean ± standard deviation or minimum-maximum range. Categorical parameters were expressed as numbers or percentages, as appropriate. For comparisons of HRCT scores among different clinical phenotypes, one-way ANOVA testing or the Kruskal-Wallis test was adopted, as appropriate. The correlations between HRCT scores and phenotypes were evaluated by Spearman’s correlation analysis. The intra- and inter-observer agreement of HRCT scores was evaluated with Cronbach’s α coefficient. If the value was higher than 0.8, the reliability was high. If the value was between 0.7 and 0.8, the reliability was good. If the value was between 0.6 and 0.7, the reliability was acceptable. If the value was less than 0.6, the reliability was poor. When the *p*-value was < 0.05, a statistical significance difference was achieved.

## Results

### Clinical characteristics of bronchopulmonary dysplasia infants

The clinical data are listed in Table 1. The clinical data of 376 cases diagnosed with BPD in the past 7 years were reviewed, and 107 infants with chest HRCT were preliminarily selected through the medical system. Of the 107 patients, 5 neonates were excluded due to the poor quality of images. Ten infants with severe pulmonary infections and congenital heart disease were also eliminated. Then, another 11 neonates were removed because their HRCT was performed after a PMA of 50 weeks. Finally, 81 infants (56 males and 25 females; GA: 28.93 ± 2.25 weeks; BW: 1335.86 ± 456.80 g) were enrolled in this study.

**TABLE 1:** Clinical data of 81 BPD patients.

Characteristic	
GA (w)	28.93 ± 2.25
>28 w	40/81
≤28 w	41/81
BW (g)	1335.86 ± 456.80
**Sex**	
Male	56/81 (69.14%)
Female	25/81 (30.86%)
Surfactant	78/81 (96.3%)
Antenatal steroids	35/81 (43.21%)
Histological chorioamnionitis	10/81 (12.35%)

BPD, bronchopulmonary dysplasia; BW, body weight; GA, gestational age.

Chest HRCT was conducted at 42.19 ± 4.82 weeks (range: 36.14–50.86 weeks) PMA. The diagnosis of PH and TBM were made at 41.72 ± 4.83 weeks (range: 34.20–50.00 weeks) PMA and 40.90 ± 4.76 weeks (range: 36.14–50.00 weeks) PMA, respectively. There was a 4.56 ± 2.82 weeks (range: 0.14–9.57 weeks) gap between the assessment of the severity of BPD and the HRCT scanning because HRCT was postponed until the conditions of the patients were stable for the examination. The time interval between HRCT and PH diagnosis was 0.48 ± 0.28 weeks. The time interval between HRCT and TBM diagnosis was 1.01 ± 0.59 weeks. The volume of the CT dose index (CTDI_*vol*_) and dose-length product (DLP) averaged 0.89 ± 0.22 mGy and 15.40 ± 5.09 mGy/cm, respectively.

### Correlation of high-resolution computed tomography scores and severity of bronchopulmonary dysplasia

A total of 17 (20.99%) infants were classified as mild BPD, 31 (38.27%) as moderate BPD, and 33 (40.74%) as severe BPD. As shown in Table 2, there were no statistical differences in GA, BW, and duration of respiratory support among the three groups. However, infants with severe BPD had significantly higher TS (*p* = 0.01), HS (*p* = 0.02), and PS (*p* = 0.02). Mosaic attenuation (*p* = 0.03), bulla/Bleb (*p* = 0.03), and linear densities (*p* = 0.01) also demonstrated higher scores in more severe BPD group with significant difference. By Spearman’s correlation analysis, TS (*r* = 0.49, *p* < 0.01), HS (*r* = 0.31, *p* < 0.01), and PS (*r* = 0.30, *p* = 0.01) were correlated with the clinical severity of BPD. Decreased attenuation (*r* = 0.21, *p* = 0.04), mosaic attenuation (*r* = 0.31, *p* = 0.01), bulla/Bleb (*r* = 0.27, *p* = 0.02), and linear densities (*r* = 0.55, *p* < 0.01) also demonstrated a correlation with BPD severity.

**TABLE 2:** High-resolution computed tomography (HRCT) scores of subgroups with different phenotypes.

BPD	Total (*n* = 81)	Mild (*n* = 17)	Moderate (*n* = 31)	Severe (*n* = 33)	*P*-value	PH (+) (*n* = 40)	PH (−) (*n* = 41)	*P*-value	TBM (+) (*n* = 20)	TBM (−) (*n* = 61)	*P*-value
GA (w)	28.93 ± 2.25	29.59 ± 2.87	29.16 ± 2.33	28.36 ± 2.95	0.27	28.23 ± 2.57	29.61 ± 2.72	0.02✩	29.15 ± 3.47	28.85 ± 2.46	0.67
BW (g)	1335.86 ± 456.80	1454.41 ± 473.97	1385.65 ± 404.51	1228.03 ± 483.83	0.19	1267.5 ± 500.1	1402.56 ± 405.29	0.19	1390.75 ± 541.59	1317.87 ± 428.96	0.54
Duration of respiratory support in hospital (d)	113.36 ± 78.28	105.59 ± 69.06	104.13 ± 98.01	126.03 ± 60.42	0.49	123.5 ± 64.18	103.46 ± 89.65	0.25	114.2 ± 96.01	113.08 ± 72.47	0.96
**HRCT scores**											
TS	18.23 ± 5.76	14.47 ± 4.95	16.94 ± 5.6	21.39 ± 4.68	<0.01✩	19.9 ± 5.02	16.61 ± 6.03	0.01✩	11.3 ± 5.04	9.98 ± 5.46	0.19
HS	10.31 ± 5.36	7.88 ± 3.48	9.68 ± 5.27	12.15 ± 5.72	0.02✩	10.65 ± 5.74	9.98 ± 5.01	0.57	8.4 ± 3.69	7.77 ± 3.56	0.34
PS	7.93 ± 3.58	6.59 ± 2.9	7.26 ± 4.02	9.24 ± 3.08	0.02✩	9.25 ± 3.11	6.63 ± 3.56	<0.01✩	19.7 ± 5.8	17.75 ± 5.72	0.50
Decreased attenuation	4.12 ± 1.98	3.82 ± 1.91	3.81 ± 1.91	4.58 ± 2.06	0.24	4.2 ± 2.05	4.05 ± 1.94	0.73	4.65 ± 1.81	3.95 ± 2.02	0.17
Mosaic attenuation	3.05 ± 2.38	2.18 ± 2.22	2.65 ± 2.26	3.88 ± 2.38	0.03✩	3.15 ± 2.36	2.95 ± 2.43	0.71	3.5 ± 2.37	2.9 ± 2.39	0.33
Bulla/Bleb	3.14 ± 2.35	1.88 ± 1.93	3.23 ± 2.09	3.7 ± 2.58	0.03✩	3.3 ± 2.33	2.98 ± 2.38	0.54	3.15 ± 2.54	3.13 ± 2.31	0.98
Linear densities	4.35 ± 1.83	3.18 ± 1.74	3.84 ± 1.9	5.42 ± 1.15	<0.01✩	5.05 ± 1.22	3.66 ± 2.07	<0.01✩	4.7 ± 1.84	4.23 ± 1.83	0.32
Consolidation	2.7 ± 2.32	2.35 ± 1.94	2.55 ± 2.32	3.03 ± 2.51	0.56	3.25 ± 2.45	2.17 ± 2.07	0.04✩	2.95 ± 2.33	2.62 ± 2.32	0.59
Bronchial wall thickening	0.15 ± 0.55	0.29 ± 0.77	0.13 ± 0.43	0.09 ± 0.52	0.46	0.15 ± 0.66	0.15 ± 0.42	0.98	0 ± 0	0.2 ± 0.63	0.17
Bronchiectasis	0.7 ± 1.41	0.76 ± 1.35	0.74 ± 1.41	0.64 ± 1.48	0.94	0.75 ± 1.51	0.66 ± 1.32	0.77	0.75 ± 1.65	0.69 ± 1.34	0.87

BPD, bronchopulmonary dysplasia; BW, body weight; GA, gestational age; HS, hyperaeration score; PH, pulmonary hypertension; PS, parenchyma score; TBM, tracheobronchomalacia; TS, total score, ✩presenting the *p* < 0.05.

### Correlation of high-resolution computed tomography scores and the diagnosis of pulmonary hypertension or tracheobronchomalacia

All the data were listed in Table 2. In total, 40 (49.38%) infants had PH, 8 in mild BPD, 13 in moderate BPD, and 19 in severe BPD. PH (+) group were born at significantly lower GA as compared to those without PH (*p* = 0.02). Higher TS (*r* = 0.28, *p* = 0.01) and PS (*r* = 0.35, *p* < 0.01) correlated with the diagnosis of PH. Among the PSs, higher scores of linear densities (*r* = 0.34, *p* < 0.01) and consolidation (*r* = 0.24, *p* = 0.03) were associated with the diagnosis of PH.

A total of 20 (24.69%) patients had TBM, 3 with mild BPD, 7 with moderate BPD, and 10 with severe BPD. Subglottic stenosis, tracheobronchial stenosis, and other large airway lesions were not found in this study. There was no significant difference in the GA, BW, and duration of respiratory support in hospitals between the two groups. In addition, there was no difference in any of the HRCT scores between the patients with or without TBM.

### High-resolution computed tomography scores among the different combinations of phenotypes

**FIGURE 1:**
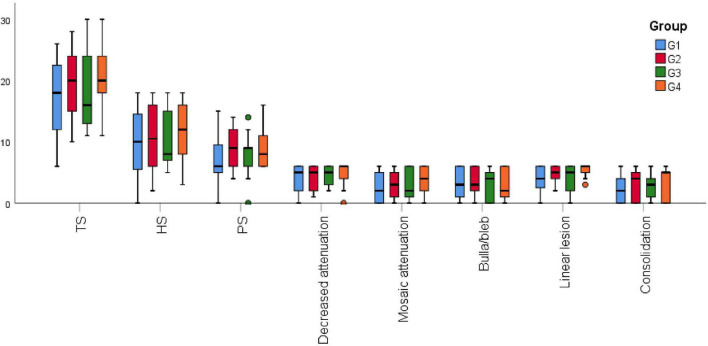
High-resolution computed tomography (HRCT) scores among the different combinations of phenotypes. G1, parenchymal disease; G2, parenchymal disease + PH; G3, parenchymal disease + TBM; G4, parenchymal disease + PH + TBM; HS, hyperaeration score; PS, parenchyma score; TS, total score.

All the patients were subdivided into four groups: parenchymal disease (Group 1 = 39), parenchymal disease + PH (Group 2 = 22), parenchymal disease + TBM (Group 3 = 9), and parenchymal disease + PH + TBM (Group 4 = 11). Group 4 had the highest TS and HS, and Group 2 got the highest PS. All the values did not have a significant difference (Figure 1).

### Intra- and inter-observer agreement

In this research, intra-observer agreement was high for HS (Cronbach’s α = 0.85), PS (Cronbach’s α = 0.90), and TS (Cronbach’s α = 0.88). Inter-observer agreement was high for HS (Cronbach’s α = 0.85), PS (Cronbach’s α = 0.86), and TS (Cronbach’s α = 0.86).

## Discussion

Bronchopulmonary dysplasia (BPD) is a heterogenous lung disease that may affect the airways and pulmonary vasculature in addition to causing lung parenchymal disease. Based on main clinical presentations, three main clinical phenotypes of BPD have been reported, namely lung parenchymal disease, pulmonary vascular disease, and airway disease (3). Classification of BPD into these clinical phenotypes may facilitate clinical management and better risk stratification of BPD (3, 19). Although HRCT is frequently used to evaluate infants with BPD, to our knowledge, this study is the first study attempting to explore the association of HRCT findings with these BPD clinical phenotypes.

Several studies have demonstrated a correlation between HRCT scores and the severity of BPD (10, 16, 20). Most researchers have categorized pulmonary abnormalities into two types: hyperaeration and parenchyma lesions. In our study, we found TS, PS, and HS were all associated with the severity of BPD. Previous studies have reported that hyperaeration lesions are the most commonly found features in infants with BPD and suggested that they represent the most sensitive structural abnormalities associated with BPD severity (15, 21). Consistent with previous data, our study found that all three subcategory scores of hyperaeration lesions (decrease attenuation, mosaic attenuation, and bulla/bleb) correlated with BPD severity. Previous studies have suggested that the severity of hyperaeration lesions is associated with obstructive lung disease and quantification of volume fraction of low attenuation regions has been used to predict impaired lung function in a patient with cystic fibrosis (22, 23). We speculate that HS may be a useful tool to predict lung function impairments and the development of obstructive lung disease in infants with BPD. Longer-term studies with pulmonary function testing will be needed to study this.

Current data suggest that pulmonary vascular disease may impact 16–25% of infants with BPD and increases the risk of mortality in these patients (3, 14, 24–28). Almost half of the patients enrolled in this study had PH, and a majority of them belonged to the severe BPD group. In our study, both TS and PS correlated with the diagnosis of PH. Among the PS scores, linear opacities were frequently observed in previous studies and are probably the most common features on HRCT images of BPD patients (20, 23). Linear opacities may be suggestive of alveolar septal fibrosis, obstructive ventilation impairment, and PH (29). In longitudinal studies, linear opacities did not change over time and might be considered irreversible damage in late BPD (22). We found in our study that linear opacity correlated with both the severity of BPD and PH. In addition, consolidation is another prominent feature in our patients and is also correlated with the diagnosis of PH. Our result suggests that taken together, linear opacities and consolidation might be useful parenchymal CT scores that might be used in the prediction of BPD severity and PH. However, further studies are needed to confirm this.

Clinical manifestations of large airway diseases in BPD patients are mainly bronchomalacia or tracheomalacia, either localized or generalized. The incidence of large airway diseases in BPD patients varies from 10 to 46% and contributes to air trapping, increased risk of respiratory infections, and prolonged positive pressure ventilation (3, 30, 31). Surprisingly, none of the HRCT findings was associated with the diagnosis of TBM. This could be possibly due to the small sample size since only 20 patients with TBM were included in this study. We did see a consistent trend of higher HS in all three sub-categories. Further studies with a larger sample size are needed to further explore the association of HRCT scores with TBM.

Chest radiography is the most commonly used imaging modality worldwide to assess the severity of lung disease in patients with BPD. However, compared with HRCT, chest radiography cannot reflect the abnormalities in the pulmonary parenchyma in detail. Therefore, it fails to accurately predict the clinical severity of BPD (15). HRCT can provide BPD patients with more objective and detailed information about pulmonary structural damages, and it has the potential to predict later symptoms and impairments. Considerable CT scoring methods have been adopted over the last 30 years to semi-quantify the structural abnormalities in BPD (12, 20). All of the methods proved abnormal CT findings in patients with BPD. However, no approach has been validated to be superior to other methods and there is no universally accepted CT scoring system now (20). In this study, the HRCT scoring system is a modified version of the widely used models in the past 10 years (10, 15–17). Compared with the Ochiai scoring system, the system developed by Sung et al. is more applicable to clinical work as it calculates the scores of each lobe, not each segment. This scoring system covers the most distinguishing features of BPD. Besides, it also presents good inter-observer and intra-observer reproducibility as shown in previous studies and also seen in our study (15, 21). We, therefore, feel that this scoring system is a good objective tool to use in the research settings.

The present study has some limitations. First, the mild BPD patients without HRCT results were excluded, which would induce selection bias. Thus, the number of abnormalities might be overestimated. Second, the sample size of this study was relatively small. In the future, multicenter research with a larger sample size should be conducted to testify to the results of this study. Third, as the tricuspid regurgitant jet is undetectable in many infants, additional echocardiographic parameters are suggested to help reflect the increased pulmonary pressure, e.g., intraventricular septum flattening, right ventricular dilation and/or hypertrophy, and right ventricular dysfunction. However, these parameters were not used in our hospitals which might underestimate the incidence of PH in this research. Fourth, the association between HRCT scores and clinical outcomes was not analyzed. Hence, this study cannot provide sufficient guidance and prognostic data for the clinical management of BPD. At last, the radiation dose of the current study is relatively high, and images were only taken at the end of expiration without angiography. Further work to reduce the radiation dose of HRCT or further exploration of the utility of ultra-low dose-controlled CT angiogram protocols is needed.

## Conclusion

High-resolution computed tomography scores are correlated with the BPD severity and PH. HS might be a useful tool in the assessment of BPD severity while linear densities and consolidation might be helpful in predicting PH.

## Data availability statement

The raw data supporting the conclusions of this article will be made available by the authors, without undue reservation.

## Ethics statement

Ethical review and approval was not required for the study on human participants in accordance with the local legislation and institutional requirements. Written informed consent from the patients’ legal guardian/next of kin was not required to participate in this study in accordance with the national legislation and the institutional requirements.

## Author contributions

QY: concept and design and acquisition of data. Q-LS: analysis and interpretation of data. QY and Q-LS: drafting the manuscript. X-HH and G-YH: revising the manuscript and final approval of the version to be published. All authors contributed to the article and approved the submitted version.
